# Optimization of Hierarchical Groove–Perforation Structures in PET Foam Cores for Wind Turbine Blade Applications

**DOI:** 10.3390/ma18122876

**Published:** 2025-06-18

**Authors:** Jinlin Li, Gaojian Lin, Xiaowei Chen

**Affiliations:** 1 School of Mechatronical Engineering, Beijing Institute of Technology, Beijing 100081, China; 3120230204@bit.edu.cn; 2 CGN New Energy Holding Co., Ltd., Beijing 100036, China; 3 Advanced Research Institute of Multidisciplinary Sciences, Beijing Institute of Technology, Beijing 100081, China; chenxiaoweintu@bit.edu.cn

**Keywords:** pet foam core, groove and perforating, optimal design, genetic algorithm, shear performance

## Abstract

To bridge the mechanical performance gap between polyethylene terephthalate (PET) foam cores and balsa wood in wind turbine blades, this study proposes a hierarchical groove-perforation design for structural optimization. A finite element model integrating PET foam and epoxy resin was developed and validated against experimental shear modulus data (α < 0.5%). Machine learning combined with a multi-island genetic algorithm (MIGA) optimized groove parameters (spacing: 7.5–30 mm, width: 0.9–2 mm, depth: 0–23.5 mm, perforation angle: 45–90°) under constant resin infusion. The optimal configuration (width: 1 mm, spacing: 15 mm, angle: 65°) increased the shear modulus by 9.2% (from 125 MPa to 137.1 MPa) and enhanced compressive/tensile modulus by 10.7% compared to conventional designs, without increasing core mass. Stress distribution analysis demonstrated that secondary grooves improved resin infiltration uniformity and interfacial stress transfer, reducing localized strain concentration. Further integration of machine learning with MIGA for parameter optimization enabled the shear modulus to reach 150 MPa while minimizing weight gain, achieving a balance between structural performance and material efficiency. This hierarchical optimization strategy offers a cost-effective and lightweight alternative to balsa, promoting broader application of PET foam cores in wind energy and other high-performance composite structures.

## 1. Introduction

Due to its excellent mechanical properties, polyethylene terephthalate (PET) foam is widely used as the core material of sandwich structures, particularly in wind turbine blades, aerospace, military, marine, automotive, and rail transportation applications [1,2]. For example, in wind turbine blades, which adopt a sandwich-structure design, PET foam is used as the shear-resisting filler material [3,4,5,6,7], effectively reducing weight due to its low density [4]. Compared to conventional materials, PET foam cores demonstrate superior performance in terms of sustainability, cost-effectiveness, and ease of processing. Specifically, PET foam cores offer higher recyclability, reduce production costs, and simplify the manufacturing process, making them an ideal choice for wind turbine blades and other industrial applications. The fiberglass-reinforced plastic (FRP) on the surface of the sandwich provides bending stiffness and the combination of the two significantly enhances the overall rigidity and shear resistance of the blades [3].

Blade sandwich structures are typically produced using a vacuum infusion process, in which the core material is bonded to the panel primarily with epoxy resin or other adhesives [8,9]. To enhance the interfacial bonding performance, the surface of the core material is often pretreated with grooves and perforations [8]. Since the elastic modulus of the adhesive is much higher than that of the core material, its penetration significantly affects the structural performance [10]. Epoxy resin, as a representative adhesive, can strengthen the core and improve its mechanical properties. However, given the higher density of the resin compared to the core material, excessive use is detrimental to lightweight design. Therefore, optimizing the groove structure on the PET foam surface is of great practical significance for enhancing mechanical performance and reducing material cost [8,10].

Various core surface pretreatment methods have been explored, including unidirectional, bidirectional, and diagonal grooves [8,11,12,13,14]. Among these, unidirectional and bidirectional grooves, along with vertical perforations, are widely used in industry due to processing convenience [8]. Xiao studied the effects of slit depth, spacing, width, and their combinations on the mechanical performance of sandwich panels without considering the weight of the core material, finding that the optimal slit depth is 2 mm less than the core thickness, slit width has minimal influence on stiffness, and reduced slit spacing significantly enhances panel stiffness [15,16]. Mostafa investigated five groove types through simulation, analyzing the impact of spacing on shear performance and identifying an optimal spacing of 45 mm [17]. Wang et al. experimentally examined the influence of orthogonal and diagonal grooves on mechanical properties and amount of epoxy resin absorption [18]. Wang et al. analyzed 16 groove configurations using the entropy weight and TOPSIS methods to determine optimal conditions based on strength, peak load, and energy absorption [19]. Ji et al. investigated the effects of groove width, hole diameter, and groove spacing on the mechanical properties of the core through experiments and numerical simulations [20].

Currently, balsa wood is widely used in the manufacturing of wind turbine blades due to its excellent mechanical properties and low weight. Its shear modulus typically exceeds 150 MPa [21,22]. Despite its cost-effectiveness, the shear modulus of reinforced PET foam cores remains inferior to that of balsa wood. To address this limitation, this study focuses on improving the shear modulus of PET foam as the primary optimization objective. By optimizing the surface groove and perforation structure, the goal is to enhance shear performance without increasing the overall weight of the core material, thereby achieving an optimal balance between mechanical strength and structural lightness.

In this study, a parametric model of PET foam core was constructed through finite element simulation, combined with optimization algorithms to assess mechanical performance under a hierarchical network groove structure. Groove spacing, width, depth, and perforation angle were selected as design variables, with shear modulus as the optimization objective. Under the constraint of constant core mass, optimal groove and perforation parameters were determined. Tensile and compressive tests were conducted on the optimized design and compared with the original structure, demonstrating significant improvements in shear and tensile and compressive properties. The comparison in Table 1 highlights the methodological differences and innovations.

## 2. Materials and Methods

### 2.1. Finite Element Model Construction and Validation

The finite element model of the existing grooved and perforated structural PET foam-enhanced core was established. The conventional enhanced PET structural core features grooves with a spacing of 30 mm, a depth of 23.5 mm, and a width of 2 mm. The perforations are spaced 30 mm apart, with a radius of 1 mm, and extend vertically through the material. According to the international standard [23], a shear specimen model with overall dimensions of 250 mm × 50 mm × 25 mm was created.

In this study, the elastic parameters of the PET and Epoxy resin materials are provided by the suppliers as shown in Table 2. The model was meshed using a hexahedral grid, resulting in a total of 71,168 cells. A constant displacement load of 1 mm/min was applied along the length of the upper surface of the model. Simultaneously, the lower surface was constrained and coupled to a reference point to facilitate the extraction and processing of the results.

**Figure 1:**
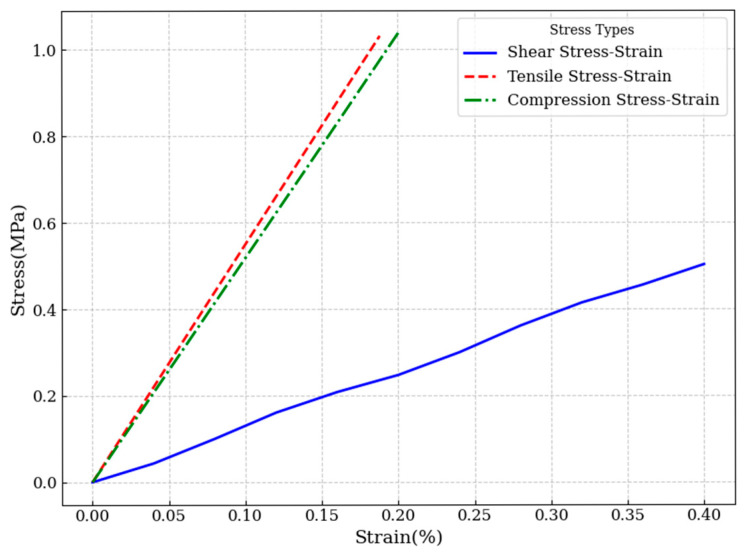
Shear and tensile stress-strain curves of the original core material.

According to international standard [23], a shear loading simulation was performed on the conventional enhanced PET foam core material. The results, as shown in Figure 1, indicate that when the transverse shear displacement reaches 1 mm, the shear reaction force at the reference point is 7234.2 N. Consequently, the shear modulus of the PET foam core is calculated to be 125.59 MPa. The FEM simulation results were validated against the experimental shear modulus of 125 MPa provided by the PET foam supplier, showing a relative error of approximately 0.47%, which demonstrates the reliability of the established finite element model.

### 2.2. Parametric Modeling and Optimal Design

#### 2.2.1. Hierarchical Network Groove and Perforating Design

**Figure 2:**
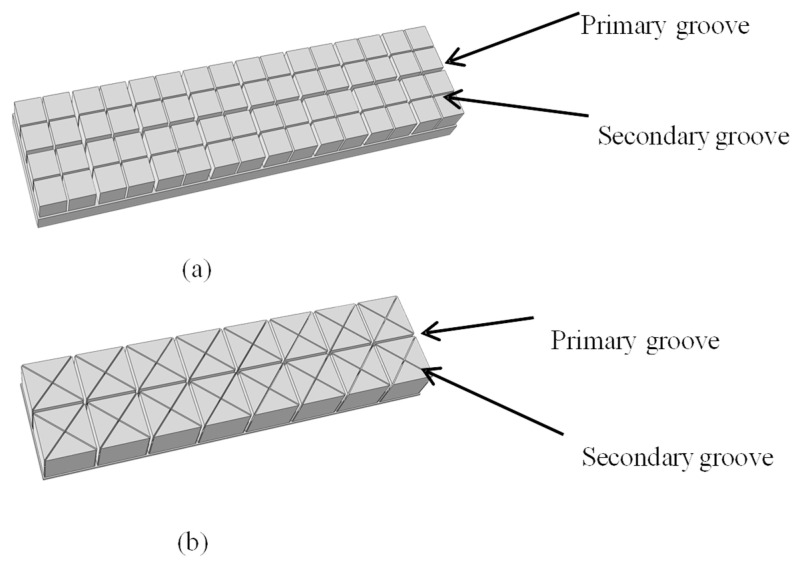
Model of hierarchical grooved core: (**a**); cross-groove; (**b**) diagonal cross-groove.

Based on the original groove structure, this study proposes a hierarchical network groove design concept aimed at enhancing the mechanical performance of polyethylene terephthalate (PET) foam core materials. Specifically, an additional level of network grooves is introduced within the existing groove structure. For clarity and consistency, the groove terminology is defined as follows:

- The primary grooves refer to the original, deeper, and wider grooves aligned along the principal load-bearing direction.
- The secondary grooves are newly introduced, shallower grooves embedded within the primary grooves to refine local stress distribution and increase structural integrity.
- As shown in Figure 2, two types of secondary grooves are investigated: the cross-groove, which is orthogonal to the primary groove, and the diagonal cross-groove, oriented at an oblique angle (typically 45°) to the primary groove direction.

**Figure 3:**
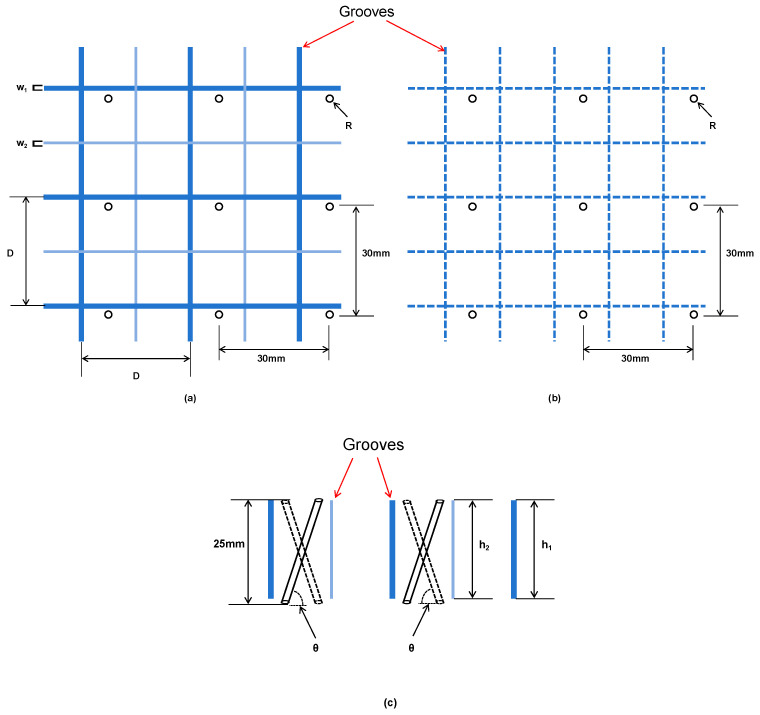
Schematic diagram of hierarchical groove and perforating: (**a**) top view; (**b**) bottom view; (**c**) side view.

As shown in Figure 3, the depth and width of the primary groove are denoted as h_1_ and w_1_, respectively, while those of the secondary groove are h_2_ and w_2_. The spacing between grooves is defined as D. At each spacing interval D, perforations are introduced, featuring a through-hole structure with a radius R and an inclination angle θ. The definitions of these variables are summarized in Table 3.

By incorporating this hierarchical groove and perforation design, the objective is to maintain or enhance the structural strength while maximizing the shear modulus and compressive resistance of the PET foam core. This structured design approach enables a more efficient transfer of loads and improved mechanical behavior under shear and compressive stresses.

#### 2.2.2. Optimization Objectives and Constraints

Shear modulus (G) is a critical indicator of a material shear performance. In this study, the shear modulus of the core material is selected as the optimization objective, with performance improvements achieved by adjusting key parameters such as groove spacing (D), groove width (w), groove depth (h), and perforating inclination angle (θ). To streamline the optimization process and reduce calculation complexity, the groove and perforating structures are treated separately, with each parameter optimized individually. This approach allows for a more efficient investigation of the effects of each parameter on the mechanical properties of the core material. The following are the constraints corresponding to the optimization of each parameter.

1. Groove Spacing Optimization

In this optimization, the amount of epoxy resin infusion is not fixed as the groove spacing is varying. Each parameter is set to its initial value: w = 2 mm, h_1_ = 23.5 mm, h_2_ = 0 mm, and θ = 90° (i.e., the original state, without the secondary groove). The groove spacing D varies from 7.5 mm to 30 mm.

As a result, the amount of epoxy resin infused will change in response to the variation in groove spacing. For the 1/4 single-cell model of the 30 mm × 30 mm groove cell, without considering the perforated structure, and under the original groove conditions (w_1_ = 2 mm, w_2_ = 0 mm, h_1_ = 23.5 mm, h_2_ = 0 mm), the original infused amount of epoxy resin (i.e., the volume of the groove cell) is:

V_0_ = (15 + 14) × 1 × 23.5 = 681.5 mm^3^ (1)

The amount of epoxy resin infused under this strategy is:

(2) $$V_{1}={\left(1\pm \frac{\pm (30-D)}{D}\right)}^{2}\cdot V_{0}$$

2. Groove Width Optimization

The mechanical properties of the structure are improved by optimizing the width of the hierarchical grooves. By reducing the width of the primary groove, the amount of resin saved is used to increase the size of the secondary groove.

- Cross-groove variable groove width optimization:

With the mold diameter fixed at D = 30 mm and primary groove depth h_1_ = 23.5 mm, the hierarchical groove configuration maintains identical widths (w_1_ = w_2_ = w). The secondary groove depth h_2_ is parametrically coupled with width w to establish an optimization framework for groove geometry. The corresponding epoxy resin infusion volume in this configuration is given by:

(3) $$V_{2}=23.5\cdot [\frac{w}{2}(30-\frac{w}{2})]+\frac{w}{2}\cdot h_{2}(30-\frac{3w}{2})$$

Assuming a constant volume of epoxy resin infusion (V_0_ = V_2_), the mathematical relationship between the groove depth (h_2_) and width (w) can be derived through geometric analysis as follows:

(4) $$h_{2}=\frac{2726-23.5w(60-w)}{w(60-3w)}$$

Applying the physical constraint 0 mm ≤ h_2_ ≤ 23.5 mm with Equation (4) yields:

1 mm ≤ w ≤ 2 mm(5)

This defines the feasible design space for groove width optimization. Systematic parametric analysis within this domain enables determination of the optimal width configuration.

- Diagonal cross-groove variable groove width optimization:

With the primary groove depth fixed at h_1_ = 23.5 mm and the secondary groove width set to w_2_ = 1 mm, the parametric relationship between the secondary groove depth (h_2_) and primary groove width (w_1_) is established. The epoxy resin infusion volume in this configuration is expressed as:

(6) $$V_{3}=23.5\cdot \frac{w_{1}}{2}(30-\frac{w_{1}}{2})+h_{2}(15\sqrt{2}-\sqrt{2}\cdot \frac{w_{1}}{2}-\frac{1}{2})$$

Under the condition of constant resin infusion volume (V_0_ = V_3_), the functional dependence of h_2_ on w_1_ is derived through geometric compatibility analysis:

(7) $$h_{2}=\frac{2726-23.5w_{1}(60-w_{1})}{60\sqrt{2}-2\sqrt{2}w_{1}-2}$$

Applying the practical constraint 0 mm ≤ h_2_ ≤ 23.5 mm to Equation (7) yields the permissible range for the primary groove width:

0.9 mm ≤ w_1_ ≤ 2 mm (8)

This defines the feasible optimization domain for the primary groove width. A systematic parametric sweep within this interval is performed to identify the optimal w_1_ value that maximizes structural performance while maintaining resin infusion requirements.

3. Groove Depth Optimization

A depth redistribution strategy is implemented by transferring resin volume from the primary to secondary grooves. With fixed parameters w_1_ = 2 mm, w_2_ = 1 mm, and D = 30 mm, the initial configuration (h_1_ = 23.5 mm, h_2_ = 0 mm) is progressively adjusted while maintaining constant resin infusion volume. The resin volume equation is formulated as:

V_4_ = 13.75 × h_2_ + 29 × h_1_ (9)

Enforcing volume conservation (V_0_ = V_4_) yields the depth correlation equation:

h_2_ = (681.5 − 29h_1_)/13.75 (10)

Subject to geometric constraints:

0 mm ≤ h_1_ ≤ 23.5 mm, 0 mm ≤ h_2_ ≤ 23.5 mm (11)

The simultaneous solution of Equations (10) and (11) defines the feasible design space:

12.357 mm ≤ h_1_ ≤ 23.5 mm, 0 mm ≤ h_2_ ≤ 23.5 mm (12)

A systematic parametric study within this domain identifies the optimal h_1_/h_2_ ratio that maximizes structural efficiency while satisfying resin volume requirements.

4. Perforation Angle Optimization

The perforation geometry is independently optimized by reallocating material volume from radial reduction to angular modification. For an initial vertical perforation (θ = 90°) with R_0_ = 1 mm, the radius-angle relationship under constant resin volume is derived as:

(13) $$R\;=\;R_{0}\surd sin\theta$$

The proposed hierarchical groove-perforation structure was developed with practical fabrication feasibility as a key consideration. The minimum groove width (w ≥ 1.0 mm) was selected to comply with common CNC milling and robotic drilling tolerances for PET foam materials. The perforation angular constraint (θ = 45–90°) prevents interference with adjacent groove features, while the width constraint ensures tool accessibility and dimensional consistency during machining.

The primary and secondary grooves can be accurately fabricated using standard industrial techniques such as CNC routing and hot-wire cutting, both of which are widely adopted in foam core shaping for composite structures. Inclined perforations can be introduced via multi-axis CNC systems or automated robotic drilling setups, which are typically available in wind turbine blade manufacturing lines.

For more complex or customized groove networks—especially in rapid prototyping or hybrid structural development—additive manufacturing approaches such as fused deposition modeling (FDM) using PETG or polyurethane-based filaments offer a flexible and precise alternative.

The proposed hierarchical groove-perforation structure was developed with practical manufacturing feasibility in mind. The minimum groove width (w_2_ ≥ 1.0 mm) was determined based on the tool diameter limitations and precision constraints of conventional CNC milling and hot-wire cutting equipment used for PET foam processing. This ensures reliable groove formation without compromising dimensional accuracy or tool stability.

Meanwhile, the inclination angle of the perforations was restricted to the range of 45° to 90°, a constraint introduced to avoid geometric interference with adjacent groove features. Perforation angles smaller than 45° may lead to overlap or unintended breakthrough into neighboring groove structures, especially in dense groove configurations, thereby compromising the integrity of the core geometry.

The primary and secondary grooves can be fabricated using CNC routing or hot-wire cutting, while angled perforations can be realized through multi-axis CNC systems or automated drilling rigs, both of which are commonly employed in industrial composite core processing lines. For complex geometries or rapid prototyping needs, additive manufacturing methods—such as fused deposition modeling (FDM) using PETG or polyurethane-based filaments—offer an alternative fabrication path with greater geometric freedom.

#### 2.2.3. Test Standards 

The test criteria and key parameters according to which the simulation calculations were performed are shown in Table 4.

#### 2.2.4. Optimization Algorithm

**Figure 4:**
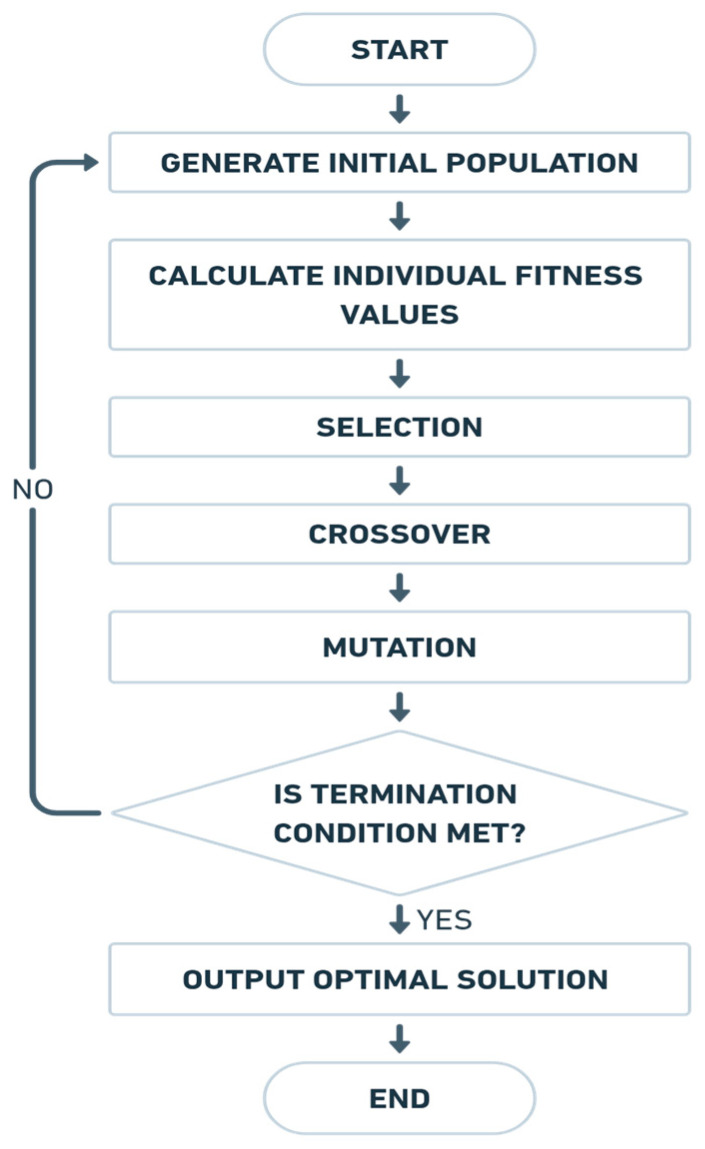
Multi-island genetic algorithm optimization process.

In this study, a Multi-Island Genetic Algorithm (MIGA) is employed for the optimization process, with the algorithm flow illustrated in Figure 4. Compared with other algorithms (e.g., PSO, DE, GA), MIGA demonstrated better global search performance and robustness in handling complex, high-dimensional design problems, making it more suitable for the optimization task in this work [26,27,28].

Building upon the traditional GA, the MIGA introduces the concept of “islands”, in which the population is divided into multiple subpopulations (islands), and individuals migrate between islands at defined intervals. This strategy effectively mitigates premature convergence to local optima and significantly enhances global search performance [29].

As shown in Table 5, in the present optimization, the algorithm is configured with five islands. Each island independently executes a GA and individual migration between islands occurs every five generations to maintain population diversity. The subpopulation size on each island is set to 10 individuals. A relatively high crossover rate is adopted to ensure the effective transmission of elite genetic information, while the mutation rate is fixed at 0.01 to balance exploration and convergence stability [30]. The algorithm is run for 10 generations, which is sufficient for convergence toward an optimal solution.

**Figure 5:**
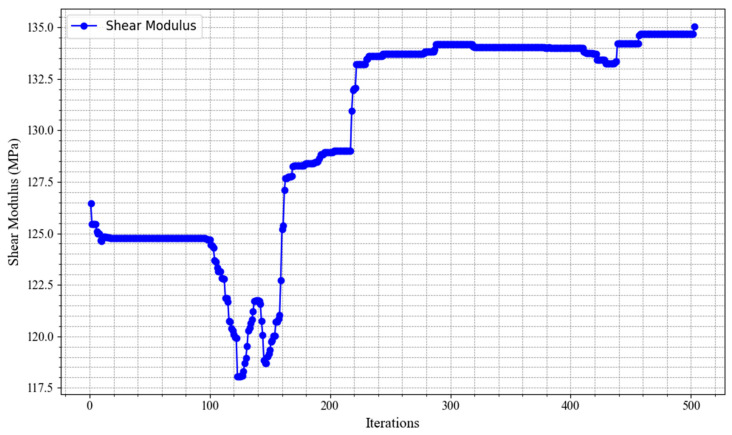
MAGA convergence process (cross-crossing groove width optimization).

Figure 5 presents the convergence behavior of MIGA in optimizing the shear modulus. The results indicate that the shear modulus increases rapidly during the early stages of evolution and reaches a global optimum after approximately 500 iterations. This demonstrates the algorithm’s efficiency and strong optimization capability. The smooth and stable convergence toward the maximum shear modulus further confirms the effectiveness of MIGA in solving complex structural optimization problems, especially in achieving global optimal solutions within a relatively small number of iterations.

## 3. Results and Discussion

### 3.1. Optimization of Hierarchical Groove Spacing

**Figure 6:**
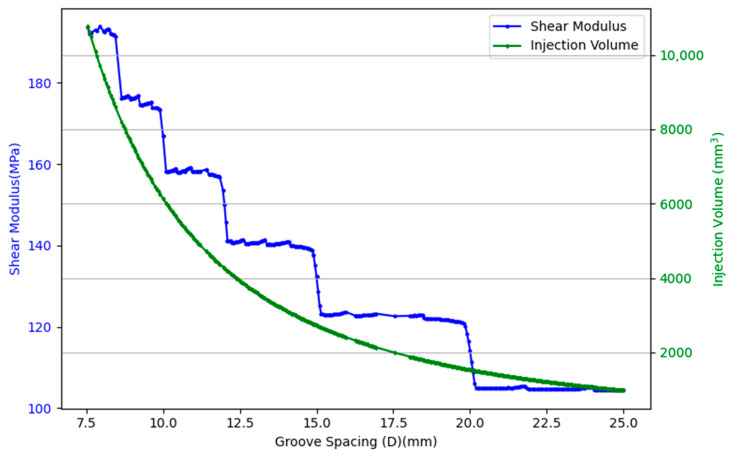
Relationship between groove spacing, shear modulus, and epoxy resin infusion volume.

As shown in Figure 6, when the epoxy resin infusion volume is not held constant, the volume of infused resin decreases nonlinearly with the increase in groove spacing. Concurrently, the shear modulus of the core material exhibits a stepwise declining trend. This behavior can be attributed to the periodic addition of two new grooves per unit area as the groove spacing changes, resulting in discrete reductions in shear stiffness. This periodic behavior stems from the discrete variation in the number of grooves per unit area. When the spacing increases or decreases beyond certain thresholds, the number of grooves in the specimen with a fixed dimension will change, resulting in abrupt transitions in shear performance. Although using a periodic boundary condition in the FEM simulation could avoid this kind of jump giving a smooth curve, here we choose to be aligned with the test standards in order to be comparable to experiments. The stair-step pattern in the shear modulus reflects the mechanical response to the structured groove density, indicating that finer groove spacing contributes to higher reinforcement but also increases resin usage. The stair-step pattern is independent of the secondary groove. Therefore, in the following optimization, the groove spacing is fixed as 25 mm.

### 3.2. Hierarchical Groove Width Optimization

**Figure 7:**
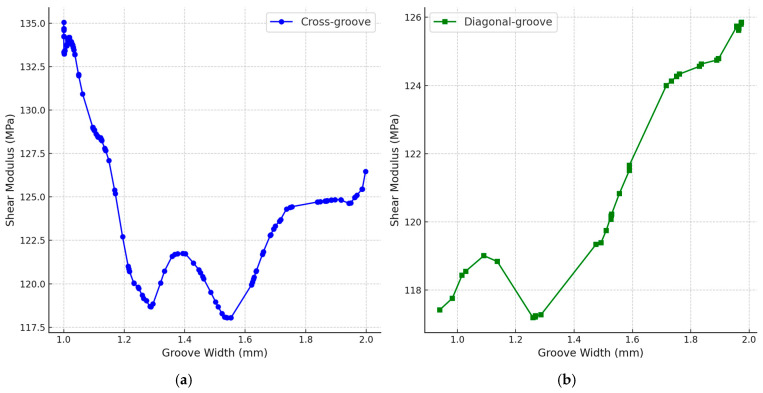
Hierarchical groove width optimization results; (**a**) cross-groove (**b**) diagonal cross-groove.

For the cross-groove configuration, as illustrated in Figure 7a, the test results indicate that the shear modulus of the core material exhibits a nonlinear response with respect to groove width. Multiple local maxima are observed, with the shear modulus reaching a minimum at approximately 1.55 mm, while a peak value of 137.14 MPa is achieved at 1 mm groove width. Compared to the original configuration with a 2 mm groove width, this represents an improvement of approximately 9.2% in shear performance, highlighting the effectiveness of width refinement in enhancing structural rigidity.

In the case of the diagonal cross-groove configuration, shown in Figure 7b, the shear modulus demonstrates a general decreasing trend as the groove width reduces from 2 mm to 1 mm. Notably, none of the optimized results within this configuration outperform the peak shear modulus achieved with the cross-groove structure. This suggests that the cross-groove configuration is more favorable for enhancing the shear performance of the core material in hierarchical groove designs.

### 3.3. Hierarchical Groove Depth Optimization

**Figure 8:**
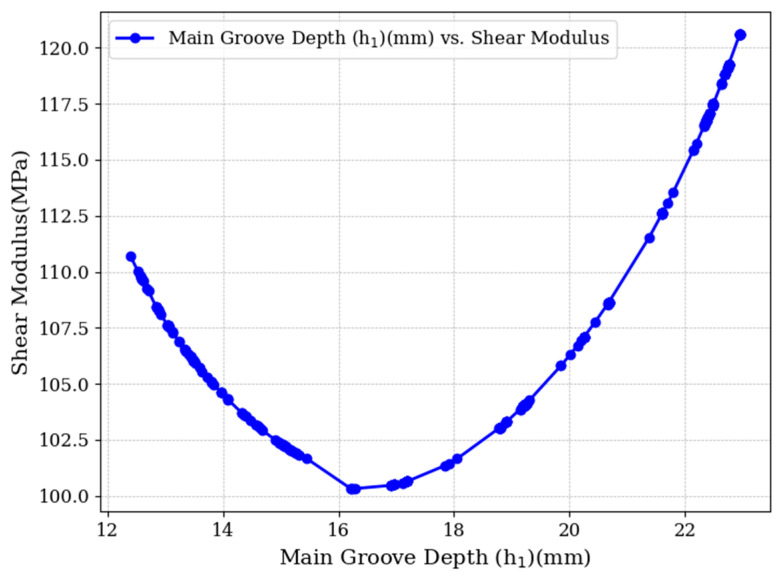
Hierarchical groove depth optimization results.

Figure 8 illustrates the effect of primary groove depth (h_1_) on the shear modulus of the core material. The results reveal a distinct nonlinear trend: as the groove depth increases, the shear modulus initially decreases, reaching a minimum value of 100 MPa at approximately 16 mm. Beyond this point, the shear modulus begins to rise again, recovering to the initial value of 125 MPa when the groove depth returns to the original design depth of 23.5 mm.

This behavior suggests that reducing the depth of the primary groove leads to a noticeable decline in shear performance. Therefore, any attempt to enhance the shear modulus by decreasing the primary groove depth is counterproductive. The initial depth configuration offers optimal structural performance in terms of shear resistance.

### 3.4. Perforating Inclination Angle Optimization

**Figure 9:**
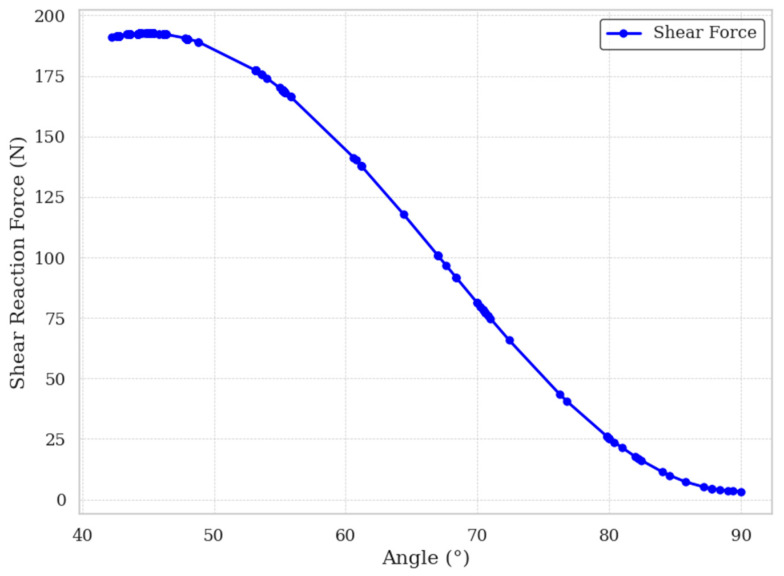
Effect of perforating angle on shear modulus.

Figure 9 illustrates the influence of different perforating angles on the shear modulus of the core material. The results demonstrate that, within the design range of inclination angles (45–90°), the shear modulus exhibits a nonlinear decreasing trend as the perforating angle increases. This indicates that, under various groove spacing configurations, a smaller perforating angle generally contributes to improved shear performance—provided that the inclined holes do not penetrate the groove structure.

To maximize the shear modulus while maintaining structural integrity, the optimal perforating angle θ is governed by its geometric relationship with the groove spacing D, groove width w, perforating radius R, and the thickness h of the core material. This relationship is given by:

(14) $$\mathrm{Tan}\theta =\frac{h}{D-w-2R}$$

Thus:

(15) $$\theta =\arctan(\frac{h}{D-w-2R})$$

Combining the optimization results of the aforementioned parameters, it is calculated that the shear modulus reaches a maximum of approximately 140 MPa when the groove width is 0.99 mm, the groove spacing is 14.85 mm, and the perforating inclination angle is 66°. However, due to machining tool limitations, the actual parameters are adjusted as follows: a groove width of 1 mm, a groove spacing of 15 mm, a secondary groove depth of 23.5 mm, a perforating angle of 65°, and a perforated hole radius of 0.9 mm.

### 3.5. Comparison of Tensile and Compression Testing for Optimized Structures

Based on the optimization results, adopting the hierarchical groove width optimization strategy combined with the cross-groove configuration significantly improves the mechanical performance of the core material. When the groove width is set to 1 mm, and the depth ratio of the primary and secondary grooves is 1:1—with both depths at 23.5 mm—the shear modulus reaches its peak value, representing the optimal mechanical configuration.

To further validate the effectiveness of the optimized design, tensile simulation experiments were conducted on the core material models before and after optimization. The tensile and compressive properties were compared by calculating the specific tensile modulus and specific compressive modulus of each configuration.

The specific modulus reflects the stiffness of a material per unit mass; a higher value indicates greater structural efficiency, offering higher stiffness at reduced weight [31]. Using the simulated support reaction force (RF), initial height (L), applied displacement (ΔL), and loaded area (A), the modulus of elasticity (E) can be expressed as:

(16) $$E=\frac{RF\cdot L}{\Delta L\cdot A}$$

The density (ρ) of the sample is determined from its measured mass and volume. Based on this, the specific tensile and specific compressive modulus are calculated using the following expression:

(17) $$\mathrm{Specific}\;\mathrm{modulus}=\frac{E}{\rho }$$

where E is the elastic modulus and ρ is the material density. This value characterizes the material stiffness per unit mass and is particularly useful for evaluating lightweight structural performance.

In the compression simulation test, the original structure exhibited a support reaction force of 10,380 N under compressive loading, resulting in a specific compressive modulus of 0.127 MPa·m^3^/kg. In contrast, the optimized structure—with a groove width of 1 mm and equal primary and secondary groove depths of 23.5 mm—achieved a specific compressive modulus of 0.140 MPa·m^3^/kg, representing an improvement of approximately 10.7% over the original design.

Similarly, tensile simulation tests were conducted on the core material models before and after optimization to assess differences in tensile performance. The original structure demonstrated a tensile support reaction force of 10,310 N, with a calculated specific tensile modulus of 0.126 MPa·m^3^/kg. In comparison, the optimized model yielded a specific tensile modulus of 0.139 MPa·m^3^/kg, marking an enhancement of approximately 10.8%.

By comparing the shear, tensile, and compressive properties of the optimized and original structures (as summarized in Table 6), it is evident that the mechanical performance of the core material has been significantly improved across all metrics following optimization.

Compared to prior studies [15,16,17,18,19,20] on PET core grooving our results show a 9.2% increase in shear modulus under a controlled resin infusion volume, supporting the feasibility of hierarchical groove-perforation structures in blade core design.

### 3.6. Mechanism Analysis of Optimization Results

The enhancement in the mechanical properties of the core material is primarily attributed to the optimized groove design. By rationally adjusting the groove width and depth, the modified structure improves the utilization efficiency of epoxy resin, leading to a more favorable resin distribution within the core. As the main load-bearing component, the resin plays a critical role in stress dispersion and load transfer under various mechanical loads such as shear, compression, and tension. The optimized groove configuration allows external forces to be distributed more uniformly, thereby enhancing the structural stability and efficiency of the core material.

**Figure 10:**
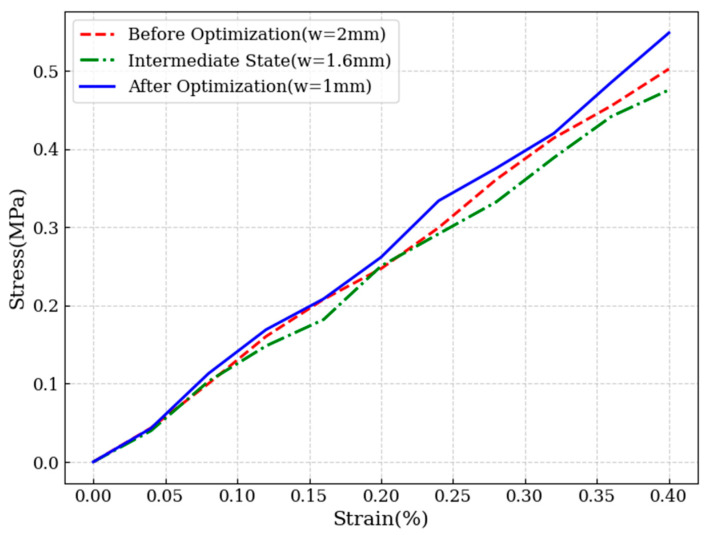
Comparison of shear stress-strain curves for different groove widths.

**Figure 11:**
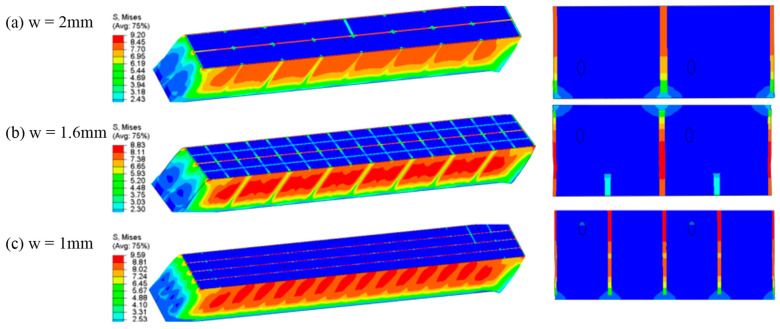
Mises stress contour for shear loading with different groove widths: (**a**) original structure; (**b**) intermediate state; (**c**) optimal structure.

To further investigate the shear performance, a comparative shear stress–strain analysis was conducted for the original structure (w = 2 mm), an intermediate state (w = 1.6 mm), and the optimized structure (w = 1 mm), as shown in Figure 10 and Figure 11. The stress cloud diagrams reveal that maximum stress concentrations occur primarily within the resin-filled regions, owing to the significantly higher elastic modulus of the resin compared to the PET foam matrix.

Although the total resin volume remains constant across all cases, the observed differences arise from the variations in resin distribution, particularly groove geometry. As the secondary groove width increases, its contribution to load bearing becomes more pronounced. A comparison between the initial and optimized structures confirms that the introduction of secondary grooves significantly improves resin utilization, thereby enhancing the overall mechanical performance of the core material.

**Figure 12:**
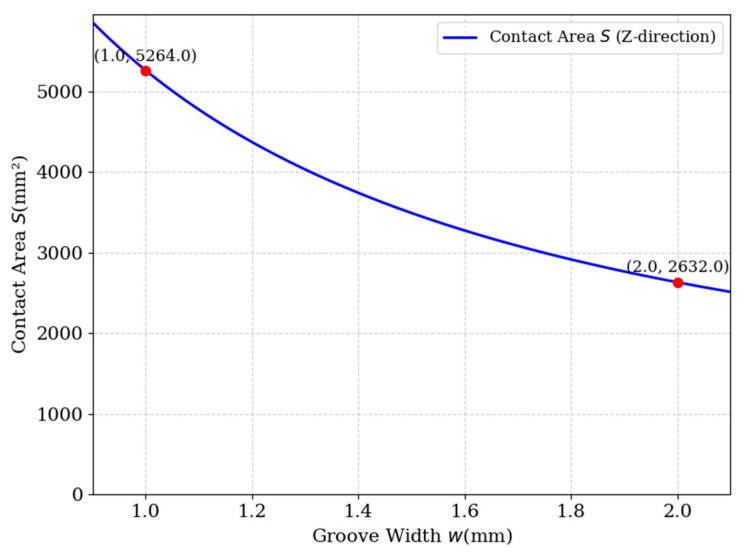
Effect of groove width on epoxy-PET foam contact area (Z-direction).

The enhancement of the stress transfer efficiency at the resin-PET interface by hierarchical groove can be quantitatively analyzed by introducing the Shear-Lag Model (SLM). According to Jones et al. [32], the relationship between the interfacial shear stress (τ_interface_) and the modulus of elasticity of the resin (E_resin_), the modulus of elasticity of the core material (E_core_), and the groove geometric parameter can be expressed as follows [32]:

(18) $$\tau_{interface}=\frac{E_{resin}\cdot E_{core}}{E_{resin}+E_{core}}\cdot \frac{w}{h}\cdot \varepsilon_{applied}$$

where w is the groove width, h is the groove depth, and ε_applied_ is the applied load strain. After optimization, the groove width w is reduced from 2 mm to 1 mm, and under the condition of constant total epoxy resin infusion, the introduction of secondary grooves effectively doubles the interfacial contact area in the cross-sectional (Z-direction) plane as shown in Figure 12, which significantly improves the load-carrying efficiency of τ_interface_. According to the theory of composite material mechanics, the distribution of resin, as a high-modulus material, has a decisive influence on the stress dispersion and load-carrying capacity of the core material. The optimized hierarchical groove design significantly improves the stress transfer efficiency by increasing the distribution path of resin. Specifically, the introduction of secondary grooves results in a more uniform distribution of resin in the core, which reduces the local stress concentration phenomenon and thus increases the overall load-carrying capacity. The Mises stress cloud from finite element simulation (Figure 11) shows that the optimized structure has a more uniform stress distribution under shear loading, and the maximum stress value increases by about 4.2%, which verifies the prediction of the theoretical model.

## 4. Fixed-Objective Optimization

Although the previously optimized core structure significantly improves mechanical performance, the target shear modulus of 150 MPa, required for large-scale blade applications, has not yet been achieved. Based on the current optimal configuration (w = 1 mm, r = 0.95 mm, θ = 65°), a fixed-objective optimization scheme is proposed to further enhance the shear modulus to meet the target threshold.

In this scheme, the perforation angle is held constant at 65°, while the radius of the perforated holes is increased appropriately to strengthen the load-transfer capability. Simultaneously, the primary and secondary groove widths are kept equal, and their depths are maintained at 23.5 mm. To achieve higher shear performance, the groove width at both levels is moderately increased to enhance the effective resin contact area and improve the stress distribution across the core.

This combined geometric adjustment—maintaining symmetry in groove dimensions and enlarging the perforation radius—aims to maximize the shear modulus while ensuring structural feasibility and manufacturing practicality.

The total epoxy resin infusion volume of the grooved and perforated structure in the original configuration was 2804.5 mm^3^. In the fixed-objective optimization, the constraint condition was set as a shear modulus ≥ 150 MPa, and the objective function was defined as the infusion volume per unit area, expressed as:

(19) $$V=\frac{25\pi r^{2}}{sin(\theta )}+23.5w(120-4w)$$

To achieve the target performance while minimizing material usage, a hybrid optimization method combining a neural network model [33,34,35,36], Bayesian optimization [35], and the genetic algorithm was employed. Discrete data within the feasible design space are generated through numerical simulations serving as the training dataset.

To ensure the reliability of the machine learning-based shear modulus prediction model, the simulation dataset was split into 80% training and 20% testing sets. Furthermore, five-fold cross-validation was applied to reduce overfitting and improve robustness. The performance of the model was evaluated using R^2^ and RMSE, yielding values of 0.999 and 0.159, respectively. These results indicate a high level of predictive accuracy across the design space, supporting the use of this model in the subsequent optimization stage.

**Figure 13:**
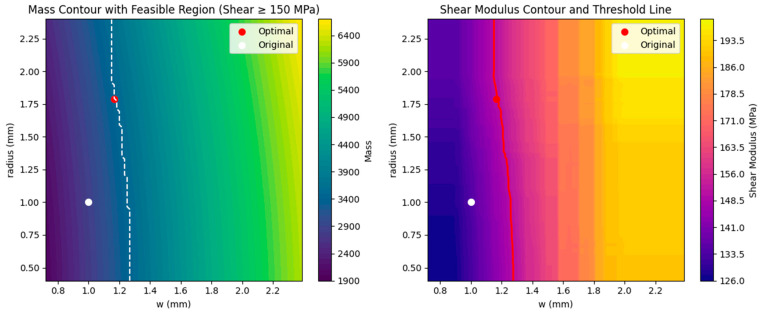
Fixed-objective optimization results of hole radius and groove width to achieve 150 MPa shear modulus.

A high-accuracy shear modulus prediction model is developed using gradient boosting trees [34], with cross-validation employed to ensure the model’s generalization capability. A constraint is imposed such that the predicted shear modulus must not fall below 150 MPa. Finally, the PSO global optimization algorithm [36,37,38] is applied within predefined parameter bounds to search for the minimal mass solution. The objective function is defined by the mass calculation formula, while the constraint function is based on the predicted shear modulus. By configuring particle swarm size and maximum iteration count, the algorithm effectively explores the parameter space to identify optimal parameter combinations that satisfy the performance constraints. The novelty of this approach lies in the seamless integration of machine learning-based predictive modeling [37] with global optimization algorithms, enabling precise and efficient optimization design of composite material parameters. The optimization results are presented in Figure 13.

The predicted optimal parameter configuration is: groove width w = 1.17 mm, perforation radius r = 1.79 mm, and perforation angle θ = 65°. Under these parameters, the predicted shear modulus is 150.1 MPa, with a minimum total infusion volume of 3448.39 mm^3^.

**Figure 14:**
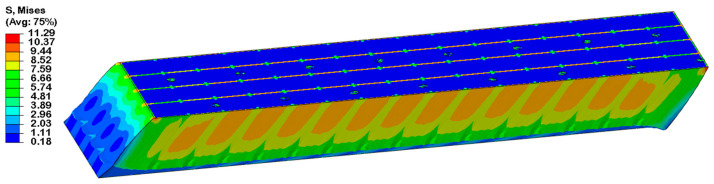
Mises stress contour of optimized models with shear modulus up to 150 MPa.

**Figure 15:**
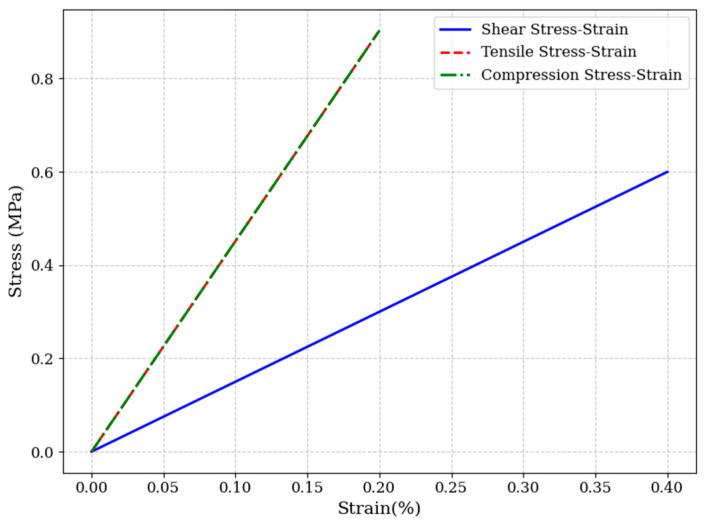
Shear and tensile stress-strain curves of optimized models with shear modulus up to 150 MPa.

As shown in Figure 14 and Figure 15, validation of the optimized design yielded an actual shear modulus of 149.94 MPa, confirming the prediction’s accuracy. This corresponds to a 20% increase in shear modulus compared to the baseline configuration, at the cost of a 22.9% increase in resin infusion volume. The result highlights a favorable trade-off between mechanical enhancement and material efficiency.

## 5. Conclusions

This paper carries out an in-depth study on the surface groove design and optimization of PET core material, describes the surface groove design method and optimization strategy, and analyzes the impact of different groove forms on mechanical properties, mainly achieving the following major results:

- An effective finite element model of PET core material is established, a hierarchical network groove method is proposed, and the study proposes an optimization scheme for a variety of core material groove parameters.
- A multi-island genetic algorithm is used to optimize the depth and width parameters of the groove on the surface of PET cores, and the optimal groove method is determined under the premise of ensuring that the total mass of the core material remains unchanged, which results in an increase of the shear modulus of the core material by 9.2%, and an increase of compression modulus and tensile modulus by about 10.7%.
- A neural network combined with a genetic algorithm was used to predict the parameter configurations for groove, resulting in an optimized core shear modulus of 150 MPa.

The hierarchical groove design proposed in this study, when applied to PET foam cores used in FRP-PET-FRP sandwich structures, can potentially improve the overall structural performance of wind turbine blades. The observed enhancement in shear modulus and compressive strength contributes directly to better load transfer and delamination resistance between the core and fiber-reinforced composite skins.

Moreover, the refined groove patterns can improve adhesive penetration and bonding strength at the core-skin interface, which is critical in high-cycle fatigue environments typical of wind turbines. By reducing local stress concentrations and enhancing energy absorption, the optimized core structure may extend the fatigue life of the blade and improve its durability under dynamic wind loading.

Potential application areas include the shear web, spar cap filler regions, and blade root reinforcements, where stiffness-to-weight optimization and mechanical robustness are of paramount importance.

## Figures and Tables

**Table 1:** Comparison of recent FEM-based groove/perforation core structure studies and the proposed optimization framework.

Study	Methodology	Performance Target	Remarks
Xiao et al. (2014–2015) [15,16]	Experimental only	Flexural, tensile strength	No FEM or optimization, no shear enhancement
Mostafa (2015) [17]	FEM parametric study	Shear stiffness, shear strength	Studied shear key spacing; no ML/data-driven optimization
Wang et al. (2022) [18]	Experimental only	Bending strength, resin usage	No FEM or groove geometry optimization
Wang et al. (2024) [19]	Experiment + Simulation	Peak load, energy absorption	No parametric optimization
Ji et al. (2022) [20]	FEM + Experiments	Shear modulus	No constraint on resin volume or manufacturability
This study	FEM + Experiment + ML-based optimization	Shear modulus, minimal resin	Fully parameterized model, data-driven, optimization

**Table 2:** Elastic parameters of the modelled materials.

	Density (Kg/m^3^)	Young’s Modulus (MPa)	Poisson’s Ratio
PET Foam	150	159	0.325
Epoxy Resin	1200	4000	0.31

**Table 3:** Definition of geometric parameters.

Symbol	Description	Unit
w_1_	Primary groove width	mm
w_2_	Secondary groove width	mm
h_1_	Primary groove depth	mm
h_2_	Secondary groove depth	mm
D	Groove spacing	mm
R	Perforation diameter	mm
θ	Perforation inclination angle	degrees

**Table 4:** Test standards and key parameters.

Items	Compression Test	Tensile Test	Shear Test
Test standard	ISO844 [24]	ASTMC297/C297M-16 [25]	ISO1922 [23]
Sample size/mm	100 × 100 × 50	100 × 100 × 50	250 × 50 × 25
Loading rate/(mm·min^−1^)	1	1	1

**Table 5:** Parameters of the multi-island genetic algorithm.

Keys	Values
Interval of Migration	5
Number of Generations	10
Number of Islands	5
Penalty Base	0.0
Penalty Exponent	2
Penalty Multiplier	1000.0
Random seed value	−1
Rate of Crossover	1.0
Rate of Migration	0.01
Rate of Mutation	0.01
Rel Tournament Size	0.5
Sub-Population Size	10

**Table 6:** Tensile and shear properties of original and optimal structural cores.

PET Foam Structure	Main Groove Depth (mm)	Depth of Secondary Groove (mm)	Shear Modulus (MPa)	Specific Modulus (Compression) MPa/(kg/m^3^)	Specific Modulus (Tensile) MPa/(kg/m^3^)
Original structure	23.5	0	125.59	0.127	0.126
Optimal structure	23.5	23.5	137.14	0.140	0.139

## Data Availability

The original contributions presented in this study are included in the article. Further inquiries can be directed to the corresponding author.

## Abbreviations

The following abbreviations are used in this manuscript:

PET	Polyethylene terephthalate
FRP	Fiberglass-reinforced plastic
TOPSIS	Technique for order preference by similarity to ideal solution
CNC	Computer Numerical Control
PETG	
ML	Machine Learning
MIGA	Multi-island genetic algorithm
SLM	Shear-lag model

## References

[1] Anthos M.X., Havalikar R.D., Antos V.T., Dey S.K., Yilmazer U. (2001). Properties and Applications of Sandwich Panels Based on PET Foams. J. Reinf. Plast. Compos..

[2] Liu X., Zhang Y., Wang L., Chen G. (2022). Research on the Feasibility of Polyethylene Terephthalate Foam Used in Wind Turbine Blades. Environ. Prog. Sustain. Energy.

[3] Xie H., Li W., Fang H., Zhang S., Yang Z., Fang Y., Yu F. (2024). Flexural Behavior Evaluation of a Foam Core Curved Sandwich Beam: Experimental Study and Numerical Simulation. Compos. Struct..

[4] Garrido A., Keymanesh M., Gibson A.G., Cheung Y.-K., Love R. (2014). Effects of Elevated Temperature on the Shear Response of PET and PUR Foams Used in Composite Sandwich Panels. Constr. Build. Mater..

[5] Steeves C.A., Fleck N.A. (2004). Collapse Mechanisms of Sandwich Beams with Composite Faces and a Foam Core, Loaded in Three-Point Bending. Part I: Analytical Models and Minimum Weight Design. Int. J. Mech. Sci..

[6] Gurit AG (2022). Core Materials Processing Guide.

[7] Cai Z., Wang L., Wu G., Ding Y., Ma J. (2020). Mechanical Behavior of Composite Materials for Innovative Wind Turbine Blades: A Review. Renew. Energy.

[8] Paris C., Tan L., Hildebrand D., Assetto M. (2020). Vacuum Infusion Process for Composite Sandwich Structures: Effects of Adhesive Viscosity and Core Perforation. Polymers.

[9] Rezaei R., Shokrieh M.M., Omidvar H., Rabczuk T. (2018). The Effect of Elevated Temperature on the Mechanical Properties and Failure Modes of GFRP Face Sheets and PET Foam Cored Sandwich Beams. J. Sandw. Struct. Mater..

[10] Wu H., Niu H., Liu M., Li H. (2023). Effect of Groove Structure on Resin Penetration and Mechanical Properties of PET Foam Core Sandwich Composite. Polymers.

[11] Steeves C.A., Fleck N.A. (2004). Collapse Mechanisms of Sandwich Beams with Composite Faces and a Foam Core, Loaded in Three-Point Bending. Part II: Experimental Investigation and Numerical Modelling. Int. J. Mech. Sci..

[12] Pan N., Gao X., Shen Y. (2021). Transverse Impact Damage and Axial Compression Failure of Square Braided CFRP/PMI Sandwich Composite Beams. Thin-Walled Struct..

[13] Pyrzowski P., Hilary J. (2020). Local and Global Response of Sandwich Beams Made of GFRP Facings and PET Foam Core in Three-Point Bending Test. Compos. Struct..

[14] Yao Z., Li X., Wang X., Zhang Y. (2020). Effect of Crystallization on Tensile Mechanical Properties of PET Foam: Experiment and Model Prediction. Polym. Test..

[15] Xiao X., Zu L., Wang J. Research on the Bending Performance of Composite Groove Sandwich Panels. Proceedings of the 20th National FRP/Composites Academic Exchange Conference.

[16] Xiao X. (2015). Effect of Surface Treatment of Core Material on Mechanical Properties of Composite Sandwich Panels. Master’s Thesis.

[17] Mostafa A. (2015). Numerical analysis on the effect of shear keys pitch on the shear performance of foamed sandwich panels. Eng. Struct..

[18] Wang S., Wang Y., Li W., Gu Y., Li J. (2022). Effects of Different Processing Forms of Core Foam on Mechanical Properties. Rubber Plast. Technol. Equip..

[19] Wang K., Yu L., Jin X., Zhang H., Yao R. (2024). Effect of Bi-Directional Groove Process of Core Material on the Bending Properties of PET Foam Sandwich Structure. J. Plast. Eng..

[20] Ji X., Qin Z., Feng W., Song X., Fu S., Wang G. (2022). Effect of Groove Hole Size on Mechanical Properties of Core Materials. Compos. Sci. Eng..

[21] Da Silva L.F.M., Kyriakides S. (2017). Mechanics of Balsa (Ochroma pyramidale) Wood.

[22] Osei-Antwi M., Castro J., Vassilopoulos A.P., Keller T. (2013). Shear Mechanical Characterization of Balsa Wood as the Core Material of Composite Sandwich Panels. Constr. Build. Mater..

[23] (2018). Rigid Cellular Plastics—Determination of Shear Properties.

[24] (2021). Rigid Cellular Plastics—Determination of Compression Properties.

[25] (2021). Standard Test Method for Flatwise Tensile Strength of Sandwich Constructions.

[26] Yan F. (2003). Genetic Algorithm for Optimal Design of Composite Laminated Structures. Shanghai Aerosp..

[27] Alba E., Tomassini M. (2002). Parallelism and Evolutionary Algorithms. IEEE Trans. Evol. Comput..

[28] Cantu-Paz E. (1999). Designing Efficient and Accurate Parallel Genetic Algorithms. Ph.D. Thesis.

[29] Gong W., Fialho Á., Cai Z., Li H. (2011). Adaptive Strategy Selection in Differential Evolution for Numerical Optimization: An Empirical Study. Inf. Sci..

[30] Xiaohua Z., Zhenwei W., Dafeng S. (2020). Parameter Optimization of Dual-Mode Power-Split Hybrid Electric Bus Based on MIGA Algorithm. J. Mech. Eng..

[31] Ashby M.F., Ashby M.F. (2011). Chapter 4—Material Property Charts. Materials Selection in Mechanical Design.

[32] Jones R.M. (1999). Mechanics of Composite Materials.

[33] Abuodeh O.R., Abdalla J.A., Hawileh R.A. (2020). Prediction of Shear Strength and Behavior of RC Beams Strengthened with Externally Bonded FRP Sheets Using Machine Learning Techniques. Compos. Struct..

[34] Malashin I., Tynchenko V., Gantimurov A., Nelyub V., Borodulin A. (2025). Boosting-Based Machine Learning Applications in Polymer Science: A Review. Polymers.

[35] Liu B., Lin H., Chen Y., Yang C. (2024). Prediction of Rock Unloading Strength Based on PSO-XGBoost Hybrid Models. Materials.

[36] Zhang X., Sun L. (2021). Optimization of Optical Machine Structure by Backpropagation Neural Network Based on Particle Swarm Optimization and Bayesian Regularization Algorithms. Materials.

[37] Berladir K., Antosz K., Ivanov V., Mitaľová Z. (2025). Machine Learning-Driven Prediction of Composite Materials Properties Based on Experimental Testing Data. Polymers.

[38] Vieira A.F.C., Filho M.R.T., Eguea J.P., Ribeiro M.L. (2024). Optimization of Structures and Composite Materials: A Brief Review. Eng.

